# Digital governance and its benchmarking college talent training under the rural revitalization in China—A case study of Yixian County (China)

**DOI:** 10.3389/fpubh.2022.984427

**Published:** 2022-10-19

**Authors:** Qinfeng Xing, Wanyan Yao

**Affiliations:** Center for Public Administration and Urban Culture, Anhui University of Science and Technology, Huainan, China

**Keywords:** rural digital governance, college talent training, rural revitalization, principal component analysis, multiple regression analysis, *SEM* structural equation analysis, Yixian County (China)

## Abstract

Rural digital governance has been the favorite subject of social governance with the aim of sustainable development of rural revitalization. Therefore, an indicator system is constructed to evaluate its satisfaction in Yixian County (China) based on principal component analysis, multiple regression analysis, and *SEM* structural equation analysis. The study finds that (1) the four dominant observation indicators, namely, “satisfaction of participation,” “satisfaction of diversification,” “satisfaction of legal norms,” and “satisfaction of technical support,” have a significant impact on the satisfaction of rural digital governance, and there is a linkage effect among the four observation indicators; (2) in terms of the four potential variables, “value guidance satisfaction” (0.772), “regulatory constraint satisfaction” (0.756), “satisfaction of support guarantee,” (0.699) and “multiple collaboration satisfaction” (0.676) have a decreasing influence on the overall satisfaction of rural digital governance, and there is much room for improvement. This study deepens the understanding of digital governance, and the following countermeasures are formed: strengthening the leading role of value concept; perfecting the constraint efficiency of “four governance” rule system; creating a new pattern of social “intelligent governance”; promoting the enabling effect of digital governance technology.

## Introduction

With the development of information technology, integrating digital technology into rural governance system to realize the rural digital governance has become a necessary means of social governance (1). Therefore, digital governance should be taken as an important starting point to strengthen the construction of digital countryside and realize the intelligent development of rural governance. Furthermore, digital governance can help rural governance break through the shackles of time and space rural to provide accurate and customized public services. In recent years, the level of rural governance has been greatly improved, and rural digital governance has given rural development with strong vitality, but there are also some urgent problems need to be solved (2). Therefore, how to effectively perfect rural digital governance becomes an important issue. Not only that, rural digital governance also has certain theoretical value and practical significance to help rural revitalization and enhance the modernization of rural governance system and governance capacity.

## Literature review

As an important narrative of social governance, scholars mostly discuss digital governance from the following aspects: First, it is the cognition of digital governance. Many scholars believed that digital governance was a key driver of changes in governments at all levels if they want to enhance transparency, accountability, and efficiency. Furthermore, they believed that digital governance was a governance concept produced after e-commerce and e-government, which is a brand-new governance model in the digital era (3, 4). Therefore, digital governance in the broad sense meant to the comprehensive management of economic and social resources with the support of information technology, but it also meant to the mode of using information technology to simplify the procedures of government administrative functions and public affairs to improve the degree of democratization in the narrow sense (5, 6). Second, it is the structure innovation of digital governance. Many scholars believed that building a digital government becomes a new structure innovation of digital governance. This is because that with this kind of structure innovation, the level of social democratization can be improved by using the resources of modern digital technology, and the construction of a modern governance system characterized can be emphasized by openness and diversity in the digital field (7, 8). Furthermore, with the help of modern information technology and its terminal technology, this kind of structure innovation can help digital government form the multi-dimensional regulation system linkage to realize a collaborative work of “good governance” organism and the reciprocity of “autonomy,” “law,” “moral,” “wisdom,” and “good governance” (9). Third, it is the operation mechanism innovation of digital governance. The world has witnessed the power of connectivity, and governments at all levels have seen the need to rethink their operation mechanism in response to this new form of connectivity. However, successfully driving the transformation process of operation mechanism innovation requires many changes as follows: a top-level design through the sharing of system thinking and digital resources, a kind of leaderships with technological knowledge, business vision, and customer orientation, and a model of “ExConomy” framework which breaks down what digital entails into three realities: customer experience is value, experimentation is necessary, and collaboration reshapes the regulatory system of digital platform (10–14).

Fruitful research achievements have been made in the field of digital governance, but theoretical discussion and few case types of research are mainly focused (15–17). However, the systematic quantitative analysis of rural digital governance is rarely involved. So the quantitative analysis of rural digital governance in Yixian County (China) is taken as the direct object in the study.

## Research design

### Study area

Yixian County belongs to Huangshan City in Anhui Province with a population of 93,500 and covers an area of 857 square kilometers. With a long history and profound culture, it is one of the core areas of the international tourism and culture demonstration zone in southern Anhui, with business cards such as “World Cultural Heritage Site” and “National Ecological Demonstration Zone.” In recent years, Yixian County has implemented the Digital Rural Development Strategy. To understand the operation situation of rural digital governance in Yixian County, it is necessary to learn from its effective experience, explore the effective operation methods of rural digital governance, and promote rural revitalization. This study conducts a case study on 14 administrative villages and communities in Yixian county (China), including Beijie Community, Guomen Community, Daxing Village, and Tuanjie Community.

### Construction of the indicator system

Based on literature review above and practical investigation in Yixian county (China), the following indicator system and its specific observation indicators are formed as follows.

#### Value guidance

Value guidance means that under the guidance of core values, value consensus should be formed to realize common goals (18). The value guidance of rural digital governance is people-centered by “good governance,” and it aims to create a new governance pattern with the goal of precise service objects, clear distribution of benefits, and timely feedback of demand. Then, the depth of digital governance can be realized. Therefore, the correctness of value guidance should be ensured, and the order and harmony of rural governance should be realized by value rationality. Based on this, the following values should be upheld.

##### First, it is participation

The governance theory is value-oriented to improve the degree of public participation. In the process of rural governance derived from social governance, it is also indispensable to stimulate the enthusiasm of people to participate in governance (19). Therefore, it is necessary to use information governance means to broaden the channels of public participation and effectively promote the public participation.

##### Second, it is facilitation

Digital governance should be committed to improve work efficiency, simplify work procedures, and promote the transformation and upgrading of participation governance mechanism with the renewal of digital governance means (20). Therefore, the network virtualization platform should be used to realize the transformation of governance “field” from real to virtual. Then, the space–time obstacles of governance can be broken to realize “flow governance.”

##### Third, it is diversification

Digital governance is inseparable from giving play to the interconnected role of various subjects and creating a new open pattern of pluralistic governance (21). Therefore, the advantages of digital governance technology should be used to effectively improve the coordination degree of social governance and improve the social fitness of rural digital governance.

##### Fourth, it is safety

Key technologies of network security are becoming more important for maintaining information security in cyberspace (22). Therefore, it is necessary to improve the application security of rural digital governance technology by the improvement of “security” system, the enhancement of risk handling capacity, and the promotion of safe development.

#### Regulatory constraint

Regulatory constraint is a kind of intervention, restriction, or constraint on the behavior of micro-subject according to relevant laws, regulations, and village culture (23). The regulatory constraint in rural digital governance is to realize the benign development of digital governance by giving full play to the restrictive role of legal norms, morality, and village culture. Regulatory constraint can control the subject's behavior at a reasonable level and effectively reduce the misunderstanding of governance. Based on this, the regulatory constraint should be highlighted in rural digital governance as follows:

##### First, it is legal norms

Legal norms are an important symbol of social progress and an important basis for the modernization of the national governance system and capacity (24). Therefore, legal norms should be guided by Xi Jinping's thought in the New Era. Then, the foundation governance can be consolidated, the long-term benefits can be stabilized, and the development path can be standardized.

##### Second, it is morality

Morality is an important symbol reflecting the essence of traditional culture in social governance mode (25). Therefore, the role of morality should be focused on the education of society, and the essence of the excellent traditional Chinese moral culture should be deeply absorbed. Then, rural digital governance can be improved to a higher moral level.

##### Third, it is village culture

Village culture is the basis of village modernity reconstruction (26). Therefore, in the process of digital embedding in rural governance, an important constraint role of village culture should be played. This is because that the development of rural digital governance relies on village culture with the aim of achieving the benign integration of village value and modern technology.

#### Multiple collaboration

Multiple collaboration refers to the collective behavior, which makes government, social organizations, and citizens in the same direction to achieve the maximum public interests in dealing with public issues (27). The multiple collaboration of rural digital governance refers to that the government, party organizations, the masses, and other subjects participate in governance together with the advantages of digital governance technology. Then, the integration of digital technology and the concept of “good governance” can be realized, and a new social governance pattern featuring “co-construction, co-governance, and sharing” can be created. Based on this, the following role should be played.

##### First, it is the party committee leadership

The party's thought should be internalized in heart and externalized in practice in the process of digital governance (28). Specifically, the role of grass-roots party organizations should be focused as a battle fortress, their command and leadership of rural governance work should be strengthened, and the enthusiasm and initiative of all departments and units should be mobilized. Then, the efficient development of rural digital governance can be realized under the guidance of party construction.

##### Second, it is the duty role of government

The government should uphold the purpose of serving the people based on earnestly performing their functions and effectively realizing their roles (29). Then, necessary support can be improved to optimize the allocation of rural digital governance resources through digital policies, action arrangements, and other means.

##### Third, it is the economic organization coordination

The development of economic organization is inseparable from the coordinated development of social value creation and commercial value production (30). Therefore, economic organizations need to enhance their innovation capacity based on promoting the transformation of digital technology and their social responsibilities.

##### Fourth, it is the intervention of NGO

The NGO has become an indispensable force in the process of socialist modernization (31). NGO, as one of behavior subjects, should actively play the role of democratic consultation to build efficient network platforms. Then, the capacity of rural digital governance can be effectively improved.

##### Fifth, it is the participation of villagers

As the main force of rural digital governance, villagers should strengthen their own awareness of participation and enhance their ability to participate (32). On the one hand, villagers should fully establish the subject consciousness, carry out self-management, self-service, self-education, and self-supervision, and actively participate in rural digital governance. On the other hand, villagers should improve the level of digital technology application and make technical preparations for participating in terms of the ability of rural digital governance.

#### Support guarantee system

The support guarantee system can consolidate the foundation and provide preconditions of rural digital governance (33). This is because that support security plays a fundamental and key role in rural digital governance. It is an indispensable part of the digital governance system. Based on this, this study forms the following views:

##### First, it is material security

Digital governance requires a lot of human, material, and financial resources. Furthermore, the completeness of the infrastructure is directly related to the effectiveness of digital governance (34). At present, the construction of rural information system should be promoted to lay a solid foundation for rural digital governance.

##### Second, it is technical support

Technical support is the physical guarantee of the hard power of digital governance (35). It is necessary to innovate the digital governance technology, strengthen the technical advantages of rural digital governance, and provide technical guarantee for the standardized, orderly, and efficient operation of rural digital governance.

##### Third, it is personnel competence

Digital governance cannot separate from the support of high-quality and professional talent team (36). Therefore, it is necessary to improve the training and introduction mechanism of talents, promote the construction of compound talent team, and constantly inject new vitality into the rural digital governance.

### Research hypothesis

Based on the above analysis, the independent variables and dependent variables required for the study are obtained and the following hypotheses are formed:

It is supposed that the four potential variables of *X*_1_, *X*_2_, *X*_3_, and *X*_4_ passed by fifteen observation indicators of *X*_11_, *X*_12_, *X*_21_……*X*_43_ are positively correlated with the dependent variables. Specific operational descriptions of the relevant constants, dependent variables, potential variables, and their observed indicators are shown in Table 1.

**Table 1:** Indicator operation assignment table.

**Indicator name**	**Indicator definition**	**Indicator description**	**Indicator assignment**
Gender	Constant	Gender involves two parameters: male, female	“Male”=“1”; “Female”=“0”.
Age	Constant	Age involves four parameters: “0–20 years old “, “21–40 years old “, “4–60 years old “, “61 years old and over”	“0–20 years old”=“1”; “21–40 years old”=“2”; “41–60 years old”=“3”; “61 years and older”=“4”.
Occupation	Constant	Occupation involves nine observation parameters: Farmers, workers, civil servants/public institutions, self-employed, entrepreneurs, students, unemployed/ retired, service personnel, others	“Farmers”=“1”; “Workers”=“2”; “Civil servants/public institutions”=“3”; “Self-employed”=“4”; “Entrepreneurs”=“5”; “Students”=“6”; “Unemployed/Retired”=“7”; “Service personnel”=“8”; “Others”=“9”.
Degree	Constant	Degree involves six observation parameters: Primary school and below, junior high school, high school, undergraduate, specialist, master and above	“Primary School and below”= “1”; “Junior High School”= “2”; “High School”=“3”; “Undergraduate”=“4”; “Specialist”=“5”; “Master,s degree and above”=“6”
Rural digital governance effect satisfaction	*Y*_0_	*Y*_0_ measured by independent variable *X*.	——
Value guidance satisfaction	*X*_1_	*X*_1_ involves four observation indicators and they are measured by Likert scale as follows: Satisfaction of participation (*X*_11_), Satisfaction of facilitation (*X*_12_), Satisfaction of diversification (*X*_13_), Satisfaction of safety (*X*_14_).	“Very dissatisfied” = “1”; “Dissatisfied” = “3”; “General” = “5”; “Satisfied” = “7”; “Very satisfied” = “9”.
Regulatory constraint satisfaction	*X*_2_	*X*_2_ involves three observation indicators and they are measured by Likert scale as follows: Satisfaction of legal norms (*X*_21_), Satisfaction of morality (*X*_22_), Satisfaction of village culture (*X_23_*).	
Multiple collaboration satisfaction	*X*_3_	*X_3_* involves five observation indicators and they are measured by Likert scale as follows: Satisfaction of party committee leadership (*X_31_*), Satisfaction of government's duty role (*X_32_*), Satisfaction of economic organization coordination (*X_33_*), Satisfaction of NGO organizations' intervention (*X_34_*), Satisfaction of villagers' participation (*X_35_*).	
Satisfaction of support guarantee system	*X*_4_	*X_4_* involves three observation indicators and they are measured by Likert scale, as follows: Satisfaction of material security (*X*_41_), Satisfaction of technical support (*X*_42_), Satisfaction of personnel competence (*X*_43_).	

### Data collection

The survey is conducted in Yixian County, Anhui Province. The questionnaires are mainly distributed in the form of combining paper questionnaires and online questionnaires. To ensure the comprehensiveness and accuracy of the questionnaire data, all the eight towns under the jurisdiction of Yixian County are taken as sample units. Furthermore, a total of 14 villages are selected by PPS (unequal probability) in the sample township. In addition, some residents in 14 villages are selected by the method of encounter sampling and judgment sampling. A total of 1,300 questionnaires are distributed and 1,180 questionnaires are effectively recovered, with an effective recovery rate of 90.77%.

Reliability is reliability, which refers to the consistency of the results obtained by using the same method to measure the same object repeatedly. The reliability analysis of the questionnaire through SPSS24.0 software (Table 2) obtained the alpha reliability coefficient is 0.823, which proves that the reliability of this questionnaire scale is acceptable.

**Table 2:** Reliability analysis.

**Reliability statistics**
Clone-on Bach Alpha	Number of terms
0.823	15

Validity refers to effectiveness, that is, the degree of things being accurately measured by measuring tools or means. After validity analysis by SPSS 24.0 software (Table 3), the KMO value of the overall questionnaire data is 0.765, between 0.7 and 0.8, and the significance level is 0.000, which proves that the validity of this questionnaire scale is good.

**Table 3:** Validity analysis.

**KMO and Bartlett test**	
Number of KMO sampling suitability quantity		0.765
Bartlett spherical test	Approximate chi- square	571.234
	Degree of freedom	105
	significant	0.000

In conclusion, the questionnaire data pass the reliability and validity tests, which have basic stability of the results for subsequent analysis.

## Methods

### Principal component analysis

Principal component analysis is a kind of analysis method for the purpose of dimensionality reduction. It is to recombine the original variables into a new set of unrelated comprehensive variables, while taking fewer sum variables according to actual needs, and reflect as much information of the original variables as possible. Principal component analysis focuses on providing a professional-theoretical explanation for the extracted common factor and each variable for data variation, so as to make a reasonable quantification of the importance of individual variables in the overall variation. The PCA combines the advantages of principal component analysis. Based on this, this study uses principal component analysis method to explore several influential key factors, so as to grasp the research focus. The specific process is got as follows:

**(1) Feasibility test**

The feasibility test is got by the original data to determine whether the original variables are correlated. According to the test results, the correlation matrix parameter KMO is 0.765, greater than 0.7. The Bartlett sphericity test statistic is 571.234 and a Sig value is 0.000, indicating that there is a significant correlation between variables, which can be further analyzed.

**(2) Extract the principal components**

In the principal component analysis, the range of load is 0–1, and the closer the load extracted from the variable is to 1, the closer the variable is to the relevant common factor. By simplifying multiple variables, the common factor variance and analysis results are obtained (Table 4). According to the data in the table, the overall effect of this factor extraction is relatively ideal.

**Table 4:** Table of common factor variance.

	**Initial**	**Extract**
*X*_11_	1.000	0.816
*X*_12_	1.000	0.612
*X*_13_	1.000	0.792
*X*_14_	1.000	0.574
*X*_21_	1.000	0.740
*X*_22_	1.000	0.713
*X*_23_	1.000	0.706
*X*_31_	1.000	0.721
*X*_32_	1.000	0.642
*X*_33_	1.000	0.669
*X*_34_	1.000	0.552
*X*_35_	1.000	0.642
*X*_41_	1.000	0.644
*X*_42_	1.000	0.622
*X*_43_	1.000	0.567

Extraction method: principal component analysis.

Principal component analysis represents the data structure with a few parameters by studying the internal dependence between many variables and observing the structural relationship in the data (Table 5).

**Table 5:** Table of total variances.

	**Initial eigenvalue**			**Extract the sum of squares of loads**		
**Composition**	**Total**	**Percentage of variance**	**Cumulative%**	**Total**	**Percentage of variance**	**Cumulative%**
1	4.468	29.786	29.786	4.468	29.786	29.786
2	2.057	13.715	43.501	2.057	13.715	43.501
3	1.310	8.730	52.231	1.310	8.730	52.231
4	1.176	7.840	60.071	1.176	7.840	60.071
5	1.001	6.673	66.744	1.001	6.673	66.744
6	0.849	5.657	72.401			
7	0.698	4.653	77.054			
8	0.668	4.452	81.506			
9	0.585	3.897	85.403			
10	0.519	3.463	88.866			
11	0.413	2.753	91.619			
12	0.397	2.650	94.269			
13	0.357	2.382	96.651			
14	0.317	2.112	98.763			
15	0.186	1.237	100.000			

**Figure 1:**
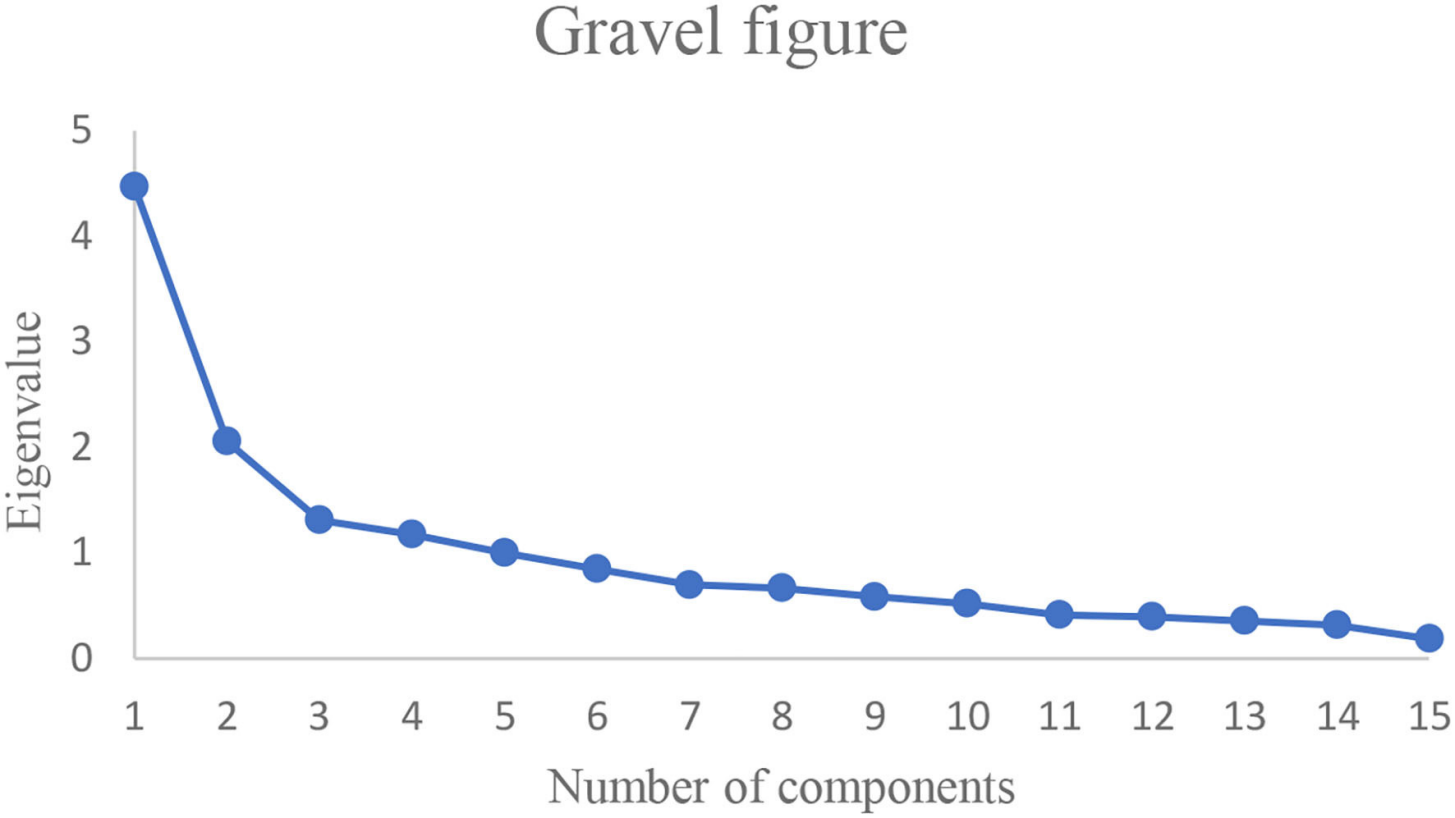
Gravel diagram.

In Table 5, the eigenvalues of components 1, 2, 3, 4, and 5 are greater than 1, which can explain 66.744% of the variance and have a better overall effect and then output the gravel diagram (Figure 1).

In Figure 1, after the confirmation of the number of factors according to the gravel map, the curve begins to flatten after the fifth factor, so that components 1, 2, 3, 4, and 5 are extracted as the principal components.

**(3) Component load analysis**

Next, the component matrix is got in Table 6.

**Table 6:** Component matrix.

	**Component**				
Observation indicator	1	2	3	4	5
*X*_11_	0.358	0.733	−0.371	−0.095	0.061
*X*_12_	0.340	0.585	−0.247	0.287	−0.102
*X*_13_	0.478	0.725	−0.118	−0.056	0.144
*X*_14_	0.599	0.331	0.275	−0.117	−0.125
*X*_21_	0.576	0.085	0.530	0.189	−0.291
*X*_22_	0.476	0.048	0.198	−0.602	−0.289
*X*_23_	0.518	0.018	0.526	−0.315	0.249
*X*_31_	0.379	0.109	0.366	0.652	−0.084
*X*_32_	0.709	−0.346	−0.103	0.057	−0.077
*X*_33_	0.534	−0.244	0.022	0.038	0.568
*X*_34_	0.638	−0.211	−0.255	−0.172	0.075
*X*_35_	0.614	−0.232	−0.355	−0.109	−0.270
*X*_41_	0.675	−0.375	−0.182	0.011	−0.123
*X*_42_	0.587	−0.097	−0.017	0.139	0.499
*X*_43_	0.543	−0.305	−0.234	0.284	−0.208

Extraction method: principal component analysis.

In Table 6, the greater the absolute value of the correlation coefficient between each observation indicator and the common factor is, the greater the influence is.

**(4) Result calculation**

Next, the weight of observation indicators is calculated by the relevant values in the total variance and component matrix tables, and then, their ranking is got in Table 7. Among them, the higher the ranking is, the greater the influence of the indicator is.

**Table 7:** Observation indicator weight and their ranking.

**Observational indicators**	**Indicator weight**	**Ranking**
*X*_11_	0.0721	1
*X*_12_	0.0685	6
*X*_13_	0.0708	2
*X*_14_	0.0664	9
*X*_21_	0.0705	3
*X*_22_	0.0673	8
*X*_23_	0.0675	7
*X*_31_	0.0659	10
*X*_32_	0.0636	12
*X*_33_	0.0611	14
*X*_34_	0.0629	13
*X*_35_	0.0587	15
*X*_41_	0.0654	11
*X*_42_	0.0697	4
*X*_43_	0.0696	5

In Table 7, the weights of *X*_11_, *X*_13_, *X*_21_, and *X*_42_ rank the top four and they need to be focused.

### Multiple regression analysis

Through principal component analysis, four observation indicators of *X*_11_, *X*_13_, *X*_21_, and *X*_42_, which have with great influence on other observation indicators, can be obtained. The influence of these four observation indicators needs to be further explored on the effect of dependent variable of rural digital governance. Since the regression analysis can establish a causal relationship between variables based on the observed data of each variable, and the satisfaction degree of rural digital governance effect is a continuous numerical variable, so the multiple linear regression statistical method is selected as follows:

First, SPSS 24.0 software is used to analyze the effect of rural digital governance as the dependent variable and the control variable as the benchmark model, and successively including *X*_11_, *X*_13_, *X*_21_, and *X*_42_, five nested models are established to, respectively, explore the influence of these indicators.

Second, the input method is selected, and “Statistics” is used by selecting “collinear diagnosis” and “Durbin-Watson.” Furthermore, “Figure” is used by selecting “ZRESID” into “Y” and “ZPRED” into “X.” Later, the SPSS software will output the operation results in Table 8.

**Table 8:** Multiple regression analysis.

**Variable**	**Model 1**	**Model 2**	**Model 3**	**Model 4**	**Model 5**
*X*_11_	-	0.404***	0.097	0.181*	0.268***
*X*_13_	-	-	0.443***	0.289***	0.164**
*X*_21_	-	-	-	0.460***	0.417***
*X*_42_	-	-	-	-	0.377***
Gender	−0.038	−0.158**	−0.016	−0.049	−0.030
Age	−0.132**	−0.097	−0.093	−0.073	−0.111**
Degree	0.265**	0.088	0.172**	0.196**	0.163**
Occupation	0.242	0.261**	0.245**	0.188**	0.146*
Constant	72.752***	58.996***	56.049***	40.047***	31.103***
Decision coefficient	0.199	0.328	0.421	0.622	0.742
Sample size	1180	1180	1180	1180	1180

^*^P < 0.10, ^**^P < 0.05, and ^***^P < 0.01.

Finally, each linear model is diagnosed as follows: The Durbin-Watson values of each model are all around 2, which means that the samples are independent; the residuals basically match the normal distribution; the VIF value is less than 5, which means there is no multicollinearity between variables. Based on the above three conditions, the regression results of each model in this study are accurate and reliable, and subsequent analysis can be conducted. Then, the following discussions can be obtained.

Model 1 shows that the overall significance test fits well and the model results are robust. Among them, the age and degree have passed the test. Based on this, variable *X*_11_ is included in model 2. *X*_11_ has a coefficient of 0.404 and significant at the confidence level of 0.01, namely *X*_11_ for each one unit raised, *Y*_0_ corresponding increase of 0.404 times. In addition, gender and occupation pass the test.

Based on model 2, variable *X*_13_ is included in model 3. *X*_13_ has a coefficient of 0.443 and significant at the confidence level of 0.01, namely *X*_13_ for each one unit raised, *Y*_0_ corresponding increase of 0.443 times. Compared to model 2, the effect of *X*_11_ has decreased in Model 3. In addition, degree and occupation pass the test.

Based on model 3, variable *X*_21_ is included in model 4. *X*_21_ has a coefficient of 0.460 and significant at the confidence level of 0.01, namely *X*_21_ for each one unit raised, Y_0_ corresponding increase of 0.460 times. Compared to model 3, the role of *X*_11_ has been improved, and the effect of *X*_13_ has decreased in model 4. In addition, degree and occupation pass the test.

Based on model 4, variable *X*_42_ is included in model 5. *X*_42_ has a coefficient of 0.377 and significant at the confidence level of 0.01, namely *X*_42_ for each one unit raised, Y_0_ corresponding increase of 0.377 times. Compared to model 4, the role of *X*_11_ has been improved, and the effect of *X*_13_ and *X*_21_ has decreased in model 5. In addition, age, degree, and occupation pass the test.

### Structural equation analysis

Structural equation model (*SEM*) is a statistical method that applies linear equation system to represent the relationship between observed indicators and potential variables, as well as the relationship between potential variables and dependent variables. The SEM structure equation is used to evaluate the overall situation of rural digital governance as follows:

First, the construction principles are formed: 1) Taking *X*_1_, *X*_2_, *X*_3_, and *X*_4_ as potential variables and the *X*_11_, *X*_12_, *X*_13_……*X*_43_ of 15 observed indicators as initial observation variables; 2) taking four relational hypotheses as the verification object; 3) the modeling is based on data obtained from case studies. Then, the AMOS24.0 software is used.

**Figure 2:**
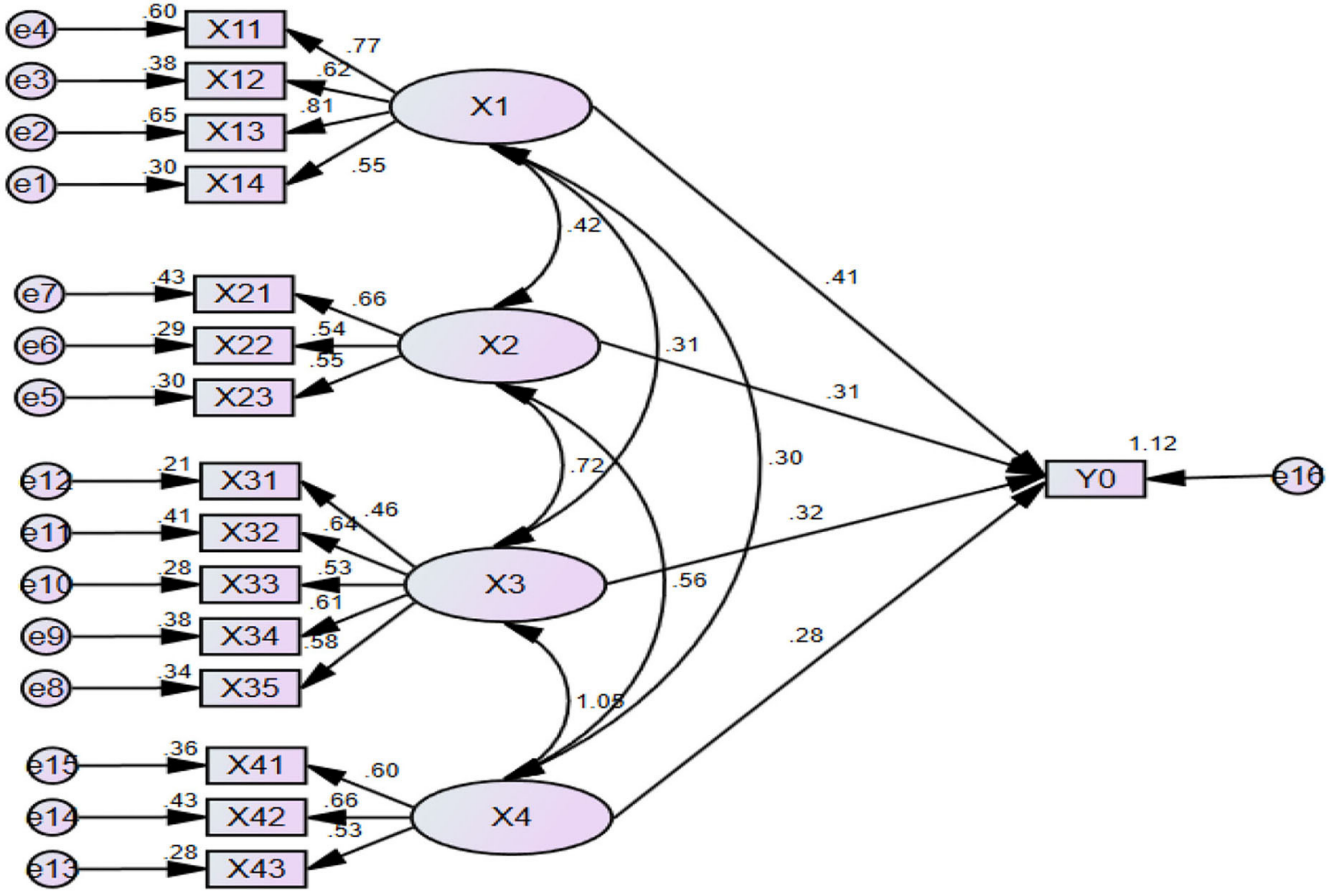
Structural equation model.

Second, according to the SEM modeling idea, the relationship among the model variables is verified and compared with the reference standard, and the nonconforming standard model parameters are modified until the indicator parameters meet the model requirements. After correction, all the revised indicators are within the acceptable range, and the model is well adapted in Figure 2.

Finally, the final modified SEM path parameters are estimated by using the AMOS 24.0 path parameter estimation function and the results are obtained (Table 9).

**Table 9:** Path coefficient.

**Path → influence**	**Standardization coefficient**	**Non-standardized coefficient**	**C.R**
*X*_1_→*Y*_0_	0.772	7.122***	6.387
*X*_2_→*Y*_0_	0.756	5.165***	5.594
*X*_3_→*Y*_0_	0.676	4.686***	5.360
*X*_4_→*Y*_0_	0.699	4.762**	5.358

**P < 0.05, and ***P < 0.01.

In Table 9, the previous hypotheses are true and the following results are formed:

First, the statistic value C.R (both greater than the standard value 1.96) and the minimum impossible event probability value P (both less than the standard value of 0.05) verify that the model path construction is reasonable and effective.

Second, *X*_1_, *X*_2_, *X*_3_, and *X*_4_ all have a significant positive impact on the dependent variable Y_0_, so the four relationship hypotheses above are valid. In addition, the influence of *X*_1_, *X*_2_, *X*_4_, and *X*_3_ on the satisfaction of rural digital governance effect decreases successively.

Third, based on the data collected form the actual investigation, the relevant values may differ from the numerical standard of the ideal data of the structural equation, which shows that there is room for improvement of some potential variables.

## Discussion

### It is necessary to highlighting key interventions to realize the linkage governance of ancient villages

Through principal component analysis and multiple regression analysis, “satisfaction of participation,” “satisfaction of diversification,” “satisfaction of legal norms,” “satisfaction of technical support,” and related logical context are sorted out, and their relative importance in the digital governance effect of Yixian County is clarified. Based on this scenario in the process of rural digital governance, modern information technology and its terminal technology should be used to strengthen the key intervention of core influence factors and realize the linkage governance of rural revitalization.

#### Stimulating the internal drive of public participation

Public participation is an important condition for the effectiveness of the rural digital governance. Digital technology can create a good environment for people to participate, in which, the exchange and sharing of information can be promoted and the efficiency of governance can be improved (37). While, if people can feel the convenience of public participation, their sense of gain and happiness can be enhanced. Then, a solid mass foundation for rural digital governance can be laid.

#### Creating a new pattern of diversified co-governance

With the semantic change from social management to social governance, the characteristics of social diversification are increasingly obvious (38). Therefore, rural digital governance should make use of the advantages of digital governance technology, highlight the characteristics of diversified co-governance, and build a cooperative governance space. Then, the development is for the people, and the fruits of development are shared by the people.

#### Improving the practice ability to the rule of law

The rule of law provides a code of conduct for rural digital governance (39). Therefore, improving the top-level design of the legal system is conducive to improving the constraint efficiency of legal norms, transforming the advantages of legal system into governance efficiency, and realizing good law and good governance (40). Then, the practice ability to the rule of law can make the rural digital governance full of vitality and harmonious and orderly.

#### Consolidating the technical foundation of “digital governance”

Digital technology is the core element of rural digital governance, which is conducive to injecting new vitality into rural development. Therefore, rural digital governance should strengthen core technology innovation. Then, the infrastructure construction can be strengthened and the foundation of digital governance can be consolidated (41). Furthermore, it is necessary to pay more attention to the digital information security issues. Only by this, the bottom line of digital security can be adhered and the information security system can be improved.

### It is inescapable to strengthen the systematic analysis to realize the holistic governance of ancient villages

Through the analysis of SEM structure equation model, it is found that the rural digital governance should not only pay attention to the unique influence of each potential variable on the dependent variable, but also connect the four potential variables from the overall perspective. Then, the system to achieve the holistic governance can be optimized.

#### Highlighting the value guidance of the Times

The influence path of value guidance ranks first. Therefore, it is necessary to pay attention to the guiding role of value concept and pay equal attention to governance technology and concept (42). Specifically, in the process of rural digital governance, not only the concept of digital governance should be updated, but also digital means should be used to optimize the effect of rural digital governance.

#### Expanding the connotation of regulatory constraints

The path influence of regulatory constraints ranks second. Therefore, we should pay attention to excavate the profound connotation of regulatory constraints (33). Specifically, in the process of rural digital governance, it is necessary to strengthen the normative role of law, enhance the binding role of morality, and highlight the value of village culture. Then, the orderly development of rural areas can be realized by improving the regulation system of rural digital governance.

#### Consolidating the cornerstone of support guarantee

The influence path of support guarantee ranks third. Therefore, the role of support guarantee should be strengthened. Therefore, in the process of rural digital governance, the training of talents should be strengthened, and core technologies should be innovated (43). Then, material guarantee can be strengthened to consolidate the foundation of rural development.

#### Strengthening the intervention of multiple collaboration

The influence path of multiple coordination ranks last, but it's important role cannot be ignored. Therefore, strengthening the collaborative situation of governance subjects is indispensable (44). Specifically, with the help of digital technology, the internal drive of various subjects can be stimulated to participate in governance (45). Then, the participation skills can be promoted to create a new pattern of “co-construction, co-governance, and shared social governance.”

## Conclusion

In view of the common problems and some heterogeneous endowments in the process of linkage governance and holistic governance, the following conclusions are formed:

### Strengthening the leading role of value concept

General Secretary Xi Jinping's thought on socialist social governance with Chinese characteristics is a scientific thinking made by the CPC Central Committee in keeping pace with the times and coordinating the overall situation at home and abroad. Among them, “implementing the national big data strategy and accelerating the construction of digital China” provide scientific theoretical guidance for rural digital governance (46). Furthermore, enabling rural revitalization with digital technology is an important way to promote the development of rural revitalization in the new era, which is conducive to the intelligent development of rural revitalization. Finally, rural digital governance should be the deep integration of value concept and digital technology, with the concept of democratic participation, interaction, and cooperation as the value goals, so that rural digital governance has both effective and temperature.

### Perfecting the constraint efficiency of “four governance” rule system

The integration mode of “four governance” is the institutional guarantee of rural digital governance, which includes “increasing vitality of autonomy, strengthening protection by rule of law, strengthening integrity by virtue, and improving efficiency by wisdom” (47). This kind mode of rural digital governance should be promoted through the development of functional modules such as “internet + village affairs” and “internet + government affairs,” to facilitate information disclosure (48). Then, the content and methods of rural digital governance can be perfected to realize civil and civilian management.

### Building a new pattern of social “intellectual governance”

The new pattern of “co-construction, co-governance, and sharing” is conducive to build a rural intelligent governance community. Among them, “co-construction” refers to the coordinated participation of multiple subjects to optimizing the division of functions (49). This is because that the optimization of this infrastructure construction can improve the relevant legal supply and combine the market forces to design a reasonable structure of digital governance. Co-governance means that each subject solves public problems or provides public services through some sustainable mechanisms (50). The optimization of these sustainable mechanisms can make all subjects give full play to the governance advantages of democratic consultation under the guidance of the government and the guidance of party construction. “Sharing” refers to the “dividend” of the governance resources, governance interests, and governance order of social governance, which are jointly maintained and transformed by the members of the society members (51). Therefore, the new pattern of “co-construction, co-governance, and sharing” is necessary to better serve rural society and the masses.

### Promoting to the enabling effect of digital governance technology

Rural digital governance technology plays a significant role in empowering governance, but its impact is also double-sided. In the information age, digital technology has created opportunities for rural governance to the diffusion effect of information technology and the universal effect of digital technology. However, “network security is very vital to national security” (52). Therefore, rural digital governance should perfect the rule of law system to improve the national network security strategy based on digital governance security system.

## Data availability statement

The original contributions presented in the study are included in the article/supplementary material, further inquiries can be directed to the corresponding authors.

## Author contributions

All authors listed have made a substantial, direct, and intellectual contribution to the work and approved it for publication.

## Funding

This work was supported by the following programs: research on the talent training mode of science and technology colleges and universities in implementing a rural revitalization strategy in Anhui Province (No. 2020jyxm0437).

## Conflict of interest

The authors declare that the research was conducted in the absence of any commercial or financial relationships that could be construed as a potential conflict of interest.

## Publisher's note

All claims expressed in this article are solely those of the authors and do not necessarily represent those of their affiliated organizations, or those of the publisher, the editors and the reviewers. Any product that may be evaluated in this article, or claim that may be made by its manufacturer, is not guaranteed or endorsed by the publisher.
